# Surface Modification of ZnO Nanorods with Hamilton Receptors

**DOI:** 10.3390/ijms16048186

**Published:** 2015-04-13

**Authors:** Lukas Zeininger, Martin Klaumünzer, Wolfgang Peukert, Andreas Hirsch

**Affiliations:** 1 Institute of Organic Chemistry, Friedrich-Alexander Universität Erlangen-Nürnberg, Henkestrasse 42, 91054 Erlangen, Germany; E-Mail: lukas.zeininger@fau.de; 2 Institute of Particle Technology, Friedrich-Alexander Universität Erlangen-Nürnberg, Cauerstrasse 4, 91058 Erlangen, Germany; E-Mails: martin.klaumuenzer@fau.de (M.K.); wolfgang.peukert@fau.de (W.P.)

**Keywords:** supramolecular chemistry, Hamilton receptor, zinc oxide, catechol, hydrogen bond, self-assembly

## Abstract

A new prototype of a Hamilton receptor suitable for the functionalization of inorganic nanoparticles was synthesized and characterized. The hydrogen bonding receptor was coupled to a catechol moiety, which served as anchor group for the functionalization of metal oxides, in particular zinc oxide. Synthesized zinc oxide nanorods [ZnO] were used for surface functionalization. The wet-chemical functionalization procedure towards monolayer-grafted particles [ZnO-HR] is described and a detailed characterization study is presented. In addition, the detection of specific cyanurate molecules is demonstrated. The hybrid structures [ZnO-HR-CA] were stable towards agglomeration and exhibited enhanced dispersability in apolar solvents. This observation, in combination with several spectroscopic experiments gave evidence of the highly directional supramolecular recognition at the surface of nanoparticles.

## 1. Introduction

**Figure 1:**
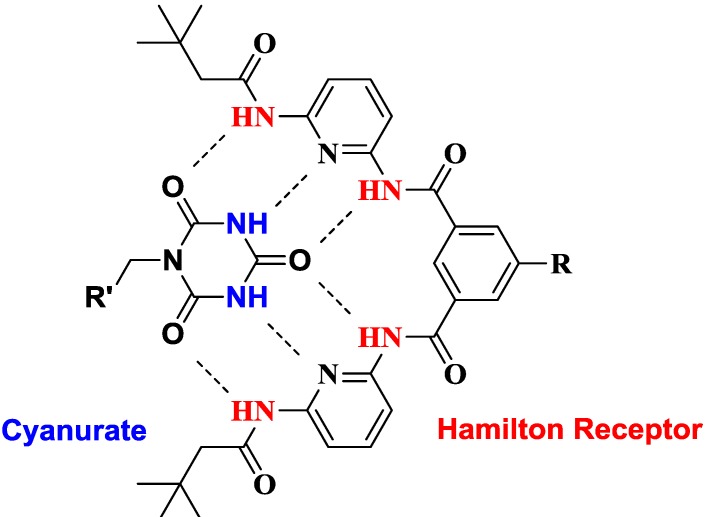
Hamilton receptor-cyanurate host-guest complex.

Supramolecular mediated self-assembly of individual building blocks represents a powerful method for both controlling implementation of organic and inorganic nanomaterials into hierarchically ordered 3D structures and generation of new nano-hybrid materials [1,2,3]. The aim to benefit from properties of thermodynamically unstable inorganic nanoparticles and to influence their characteristics through surface modifications is determined by stabilization of nano-sized matter [4]. A dense coverage of the surface through chemical functionalization leads to inhibition of Ostwald ripening [5]. However chemical transformations based on substitution reactions of covalently bound ligands on the particle surface can be problematic because desorption of the initial ligand creates a defect in the shell providing highly reactive surfaces, which can promote irreversible changes of the nanoparticles like agglomeration and growth [6]. A combination of stable covalent surface attachment with the possibility of reversible modification of particle properties is accomplished by the concept of supramolecular self-assembly of surface bound ligands with functionalities [7,8] Such an approach combines the advantages of both, avoiding the tendency of nanoparticles to agglomerate with the potential of reversible attachment of specific guests towards the generation of hybrid architectures. Among other supramolecular forces, dynamic hydrogen bonding-based systems are used for the non-covalent association of individual modules [9]. Such strategies involve the formation of ordered nanoparticle aggregates employing polymers [10], biomacromolecules, such as DNA [11] and proteins [12]. A well-established artificial hydrogen bonding motif is the Hamilton receptor (Figure 1), which can easily and strongly bind cyanuric and barbituric acid derivatives due to six complementary hydrogen bonding interactions [13].

**Figure 2:**
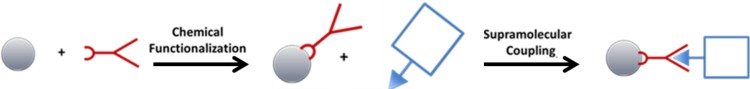
Concept of Hamilton receptor (red) mediated self-assembly of cyanurate functionalities (blue) on the surface of nanoparticles (grey).

In such a highly specific, rigid and directional host-guest complex association constants range between 10^3^ and 10^6^ M^−1^ in apolar solvents [14,15]. Thus, many supramolecular architectures employing this key-lock principle were investigated, including self-assembled dendrimers [16,17,18], polymers [19,20], self-assembled monolayers [21,22], dynamic systems [23,24] and charge-transfer architectures [25,26,27,28,29]. Recently, we could demonstrate a successful functionalization of single-wall carbon nanotubes with the Hamilton receptor [30]. However, the covalent grafting of inorganic nanoparticles with such receptors for the detection of specific organic cyanurate/barbiturate functionalities has not been described so far. Such a construction represents a unique approach for the self-assembly of individual modules towards organic-inorganic hybrid architectures. In this paper we describe, for the first time, a chemical functionalization of nanoparticles with Hamilton receptors and subsequent noncovalent attachment of organic cyanuric acid derivatives (Figure 2).

We report on the synthesis of a hitherto unknown Hamilton receptor **4** covalently equipped with a catechol anchor group, suitable for the binding to metal oxides, in particular zinc oxide [31,32]. One-dimensional zinc oxide nanorods [ZnO] were synthesized and used for surface functionalization, because they provide good stability at the nanoscale, are intensively investigated and as wide-bandgap semiconductor material relevant for many applications in electronics and optoelectronics [33,34]. The particles were coated in a wet-chemical process with the Hamilton receptor to afford monolayer-grafted nanorods [ZnO-HR]. In a detailed characterization study the conditions for stable monolayer grafting were determined. The hybrid material [ZnO-HR] featured stability against agglomeration and growth through stable monolayer surface functionalization and was accessible for further noncovalent surface modification. The unique concept was applied for the formation of newly organized organic-inorganic hybrid architectures. The coupling of tailor designed alkyl-ester substituted cyanurates **5** was accomplished to yield the hybrid structures [ZnO-HR-CA], which featured enhanced dispersability of the ZnO nanorods in apolar solvents.

## 2. Results and Discussion

### 2.1. Synthesis of Building Blocks

**Scheme 1:**
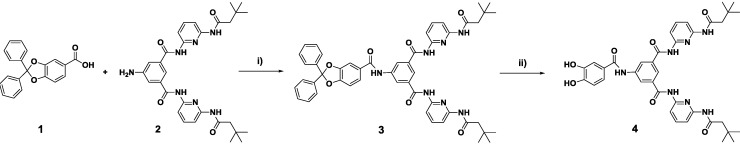
Synthesis of Hamilton receptor **4**; (i) EDC, HOBt, DMF, 0 °C − rt, 20 h, yield: 82%; (ii) TFA, rt, 24 h, yield: 99%.

A Hamilton receptor covalently equipped with a catechol anchor group suitable for the functionalization of zinc oxide surfaces was prepared as outlined in Scheme 1. First, an amine-Hamilton receptor **2** was synthesized according to a literature procedure [16]. A coupling of this amine **2** with protected protocatechuic acid **1** under modified Steglich conditions using 1-(3-dimethylaminopropyl)-3-ethylcarbodiimide (EDC) and 1-hydroxybenzotriazole (HOBt) as coupling agents resulted in Hamilton receptor derivative **3** covalently linked to the catechol anchor. Column chromatography on silica was required to isolate highly pure material. Since catechol has a strong affinity for the attachment to SiO_2_, the dihydroxy functionality had to be protected. We chose the diphenylmethane-protecting group, as it can be removed very easily by treating with trifluoroacetic acid at room temperature, resulting in a quantitative conversion to the target molecule **4**.

**Figure 3:**
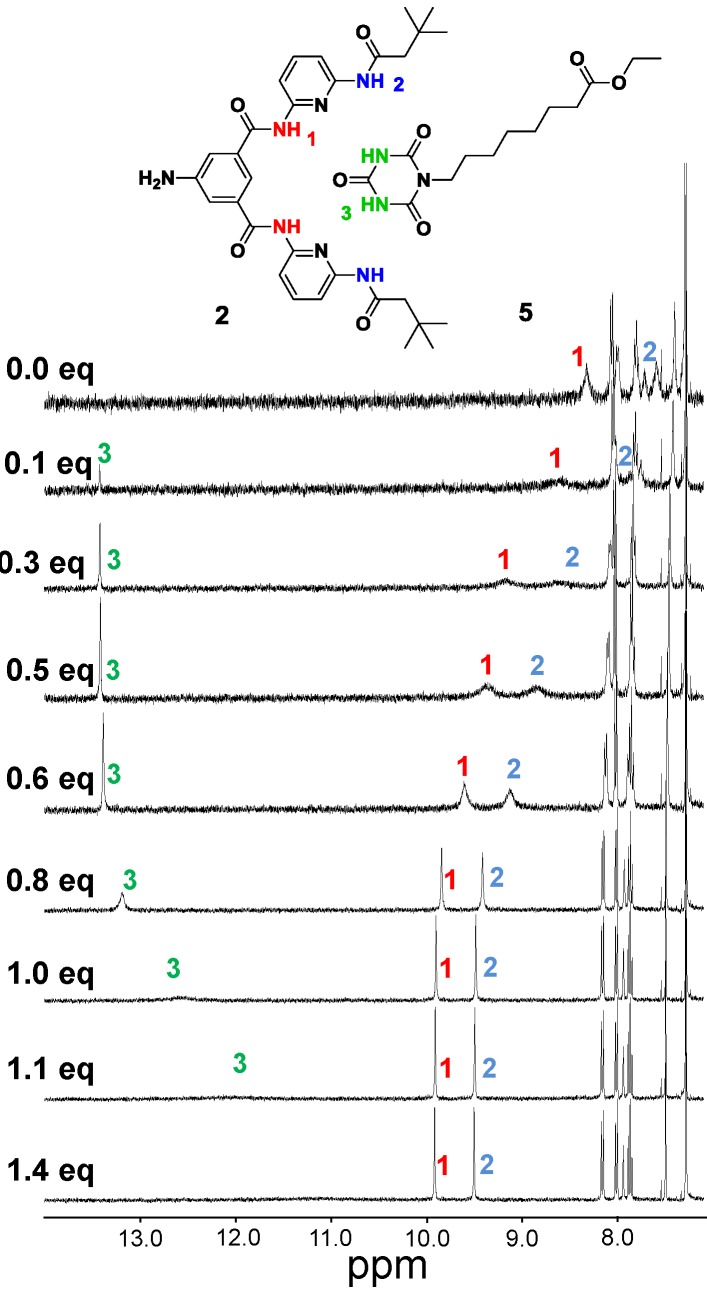
Binding motif between Hamilton receptor **2** and cyanurate **5** with indication of the NH protons NH1, NH2 and NH3 and ^1^H-NMR (400 MHz) spectra of **2** at 2.0 mM in CDCl_3_ with various equivalents of **5**.

As suitable building block for the formation of a hydrogen bond complex with Hamilton receptors cyanurate **5** was synthesized by nucleophilic substitution of cyanuric acid with ethyl 8-bromooctanoate under basic conditions. The alkyl ester moiety was chosen for solubility reasons in chloroform. For the determination of the binding affinity of this particular host guest complex, we titrated amine-Hamilton receptor **2** with equivalents of cyanurate **5**. A chemical shift of the NH-protons which are involved in the hydrogen-bonding complex in the ^1^H-NMR spectra (Figure 3) was observed. The amine-Hamilton receptor **2** was used to avoid any competing hydrogen bonding interactions with the catechol moiety in **4**. By plotting the shifts of the internal NH-protons of the Hamilton receptor *vs.* the titrated equivalents of cyanurate the association constant for this complex was calculated to be 4.1 × 10^4^ M^−1^, using the software Hyp NMR 2008^®^ (version 4.0.68; Protonic software, Leeds, UK).

As metal oxide core system, one dimensional zinc oxide nanorods [ZnO] were synthesized according to procedures reported earlier [33]. The ZnO nanorods [ZnO] of up to 90 nm in length and up to 20 nm in diameter were obtained by precipitation of zinc acetate dihydrate, Zn(OAc)_2_·2H_2_O with potassium hydroxide, KOH in methanol and subsequent refluxing for 49 h at 65 °C. X-ray diffraction (XRD) and high resolution transmission electron microscopy (HRTEM) confirm the presence of ZnO nanorods with its hexagonal Wurtzite structure (B4-type) that are elongated along the crystallographic *c*-axis. The specific surface area of [ZnO] was determined by BET analysis to be 19.76 m^2^/g.

### 2.2. Chemical Surface Functionalization

**Figure 4:**
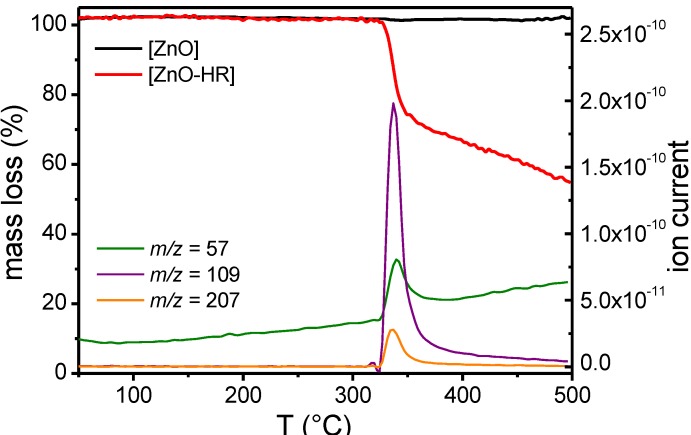
TGA-MS curves of pure and functionalized ZnO nanorods. The ion currents belong to the TGA curve of the functionalized particles.

The nanoparticles [ZnO] were initially coated in a wet-chemical process with catechol Hamilton receptor **4** to afford the hybrids [ZnO-HR]. The particles were functionalized from 0.15 wt % dispersions in methanol. After sonication and centrifugation all samples were washed three times before analysis. A distinct decrease of Hamilton receptor absorption after functionalization was observed in UV-Vis analysis of the supernatant, indicating surface adsorption. Next, TLC plate experiments revealed interactions of [ZnO] with Hamilton receptor **4**. While pure Hamilton receptor **4** was eluted, a mixture of **4** with [ZnO] stayed at the baseline. In initial experiments, the nanorods [ZnO] were exposed to 25.0 mM Hamilton receptor solutions. Such high amounts of ligands, added far above stoichiometric conditions in respect to monolayer surface coverage resulted in multilayer functionalized nanoparticles, as followed from a TGA-MS experiment (Figure 4). There are two factors that explain such an observed multilayer formation. First, Hamilton receptors are very likely to form hydrogen bonding aggregates and second catechol is known to form multilayers if applied in too high concentrations during the functionalization process [31,35]. However, the high amount of adsorbed Hamilton receptor enabled a detection of mass fragments in a TGA-MS experiment (Figure 4). In the region of weight loss of the functionalized sample [ZnO-HR] at ~350 °C, simultaneously measured mass spectra revealed particular peaks of ion current for *m*/*z* = 57, 109 and 207. These mass fragments could be assigned to fragment ions of **4** (Hamilton receptor arm). In contrast, no notable weight loss was observed when measuring the pure nanorods [ZnO].

For the purpose of attaching cyanurates directly to surface bound Hamilton receptors, monolayer functionalized nanoparticles were required. The findings outlined above suggested that concentrations during functionalization needed to be decreased. To determine the requirements for complete monolayer surface coverage the particles were now exposed to concentrations from 0.1 to 1.5 mM. The obtained hybrids [ZnO-HR] were analyzed in TGA measurements. The weight loss for each sample was converted into grafting density employing the equation [36] (with θ: grafting density; wt: mass loss; *M*_W_: molecular weight; SSA: specific surface area):

(1) $$\text{θ}=\;\left(\frac{\text{wt}}{\text{100}-\text{wt}}\right)(\frac{6.022\times 10^{23}}{M_{\text{W}}\times \text{SSA}})$$

**Figure 5:**
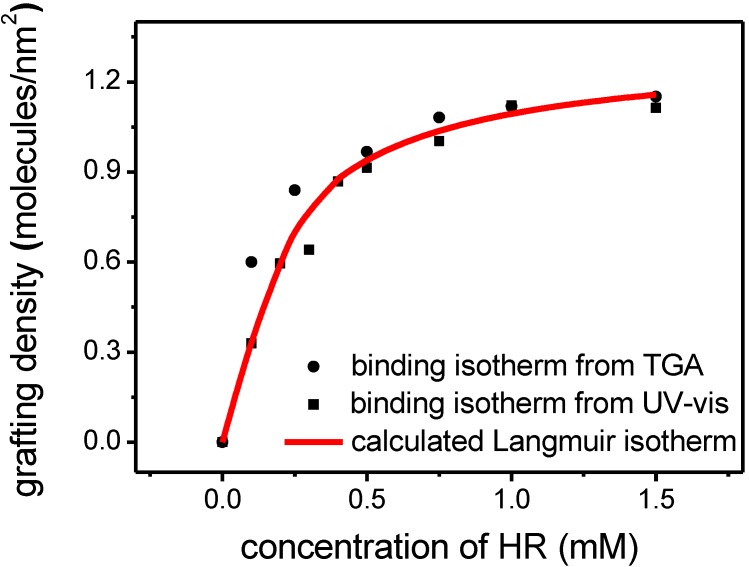
Experimentally determined grafting densities for [ZnO-HR] with different concentrations of **4** (from TGA and UV-Vis) and calculated Langmuir isotherm.

A plot of grafting density *vs.* concentration resulted in a typical Langmuir isotherm for monolayer adsorption (Figure 5). By converting the data points into a linear plot (1/θ *vs.* 1/c) a linear fit of the experimental results could be performed. From the resulting equation a specific adsorption constant for this monolayer functionalization was calculated to be 11.5 × 10^3^, describing the equilibrium between ZnO nanorods, the Hamilton receptor molecule and the solvent methanol (calculation in detail in ESI). As another result the theoretical maximum monolayer grafting density for this molecule was determined to be 1.1 molecules/nm^2^, which is consistent with previously reported grafting densities of catechols on zinc oxide [8,31]. Through insertion of these both values into the Langmuir-equation, we obtained a calculated binding isotherm (Figure 5). These systematic binding experiments revealed that applying concentrations of 0.75 to 1.5 mM provided monolayer coverage.

**Figure 6:**
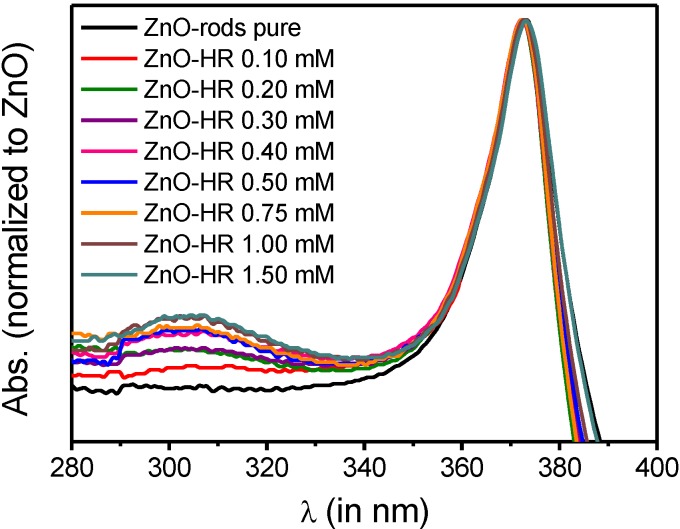
UV-Vis spectra of [ZnO] and [ZnO-HR] (particle concentration: 0.15 wt %).

UV-Vis measurements confirmed the results obtained from TGA analysis. Normalized UV-Vis spectra (Figure 6) of functionalized samples [ZnO-HR] (normalized to ZnO bandgap absorption at λ = 360 nm) displayed an increased absorption maximum at λ = 302 nm, caused by adsorbed Hamilton receptor **4**.

Although disregarding the absorption variation due to light scattering effects it was feasible to calculate the amount of adsorbed Hamilton receptor. The difference between absorption of functionalized [ZnO-HR] and non-functionalized nanorods [ZnO] was accounted to the absorption of adsorbed **4**. Employing the following formula grafting densities were obtained, which were seen to be in good accordance with the results from TGA analysis (with θ: grafting density; ε: extinction coefficient; V: volume and d: diameter of UV-cuvette; SA: surface area of ZnO rods in cuvette):

(2) $$\text{θ }=\;\left(\frac{\left({\text{Abs}}_{\text{302nm}}-{\text{Abs}}_{\text{302nm}}\left(\text{ZnO}\right)\right)\;\times \text{ V}}{\text{ε }\times \text{ d}}\right)(\frac{6.022\;\times \;10^{23}}{\text{SA}}\text{)}$$

**Figure 7:**
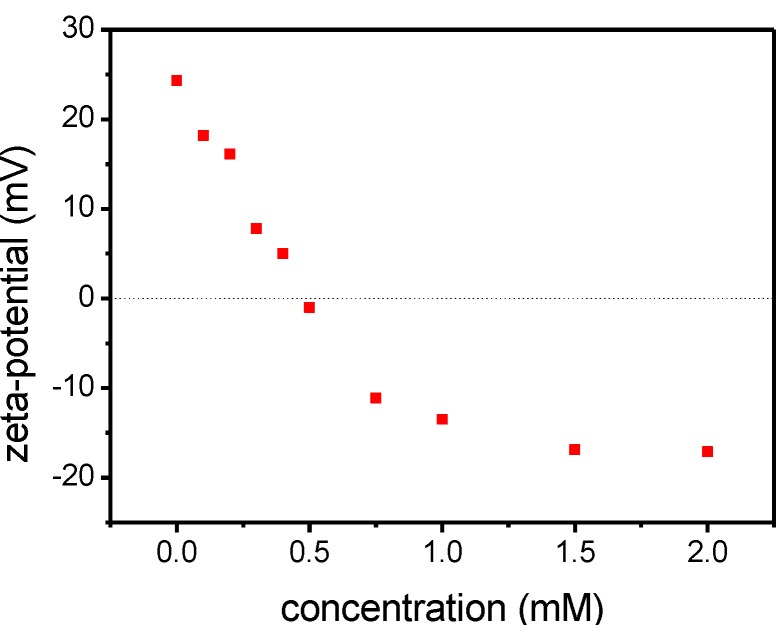
Zeta-potential trend of [ZnO-HR] functionalized with different concentrations of **4** in methanol.

Finally, the functionalization with different concentrations of **4** was tracked in zeta-potential measurements. A zeta-potential of +24.3 mV was measured for [ZnO]. The zeta-potential decreased depending on the concentration added during the functionalization process as depicted in Figure 7. Dispersions of samples, functionalized with a concentration leading to a zeta-potential near the isoelectric point (0.3–0.6 mM), were unstable in methanol. For monolayer-functionalized particles a value of −17.1 mV was reached. The trend confirmed the saturation behavior between 0.75 and 1.50 mM. The monolayer functionalized hybrid systems [ZnO-HR] were stable against any growth processes, even after drying.

### 2.3. Supramolecular Self-Assembly

**Figure 8:**
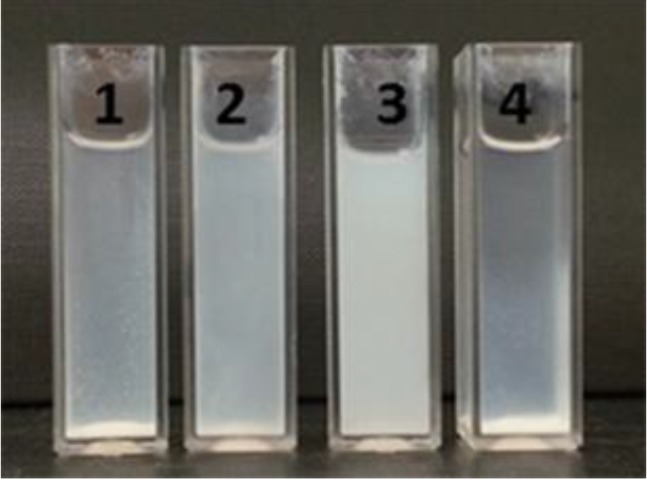
Dispersions of [ZnO] (**1**), [ZnO-HR] (**2**) and [ZnO-HR-CA] (**3**) in chloroform and [ZnO] in a 1.0 mM cyanurate **5** solution in chloroform (**4**) after 8 h (particle concentration: 0.15 wt %).

The supramolecular binding on the surface was accomplished by adding cyanurates in chloroform. Prior to the addition, [ZnO-HR] were transferred into the apolar solvent through centrifugation of the dispersions and subsequent redispersion in chloroform. Then, cyanurate **5** was added in 1 mM concentration to yield the final bilayer coated nanoparticles [ZnO-HR-CA]. The addition was accompanied by individualization of the coated nanoparticles and, therefore, affected the stability of the nanoparticle dispersions. An improved dispersability of [ZnO-HR-CA] resulted because (i) the steric stabilization of the nanoparticles was improved through the alkyl ester moiety and (ii) mainly due to the formation of the highly directional six-fold hydrogen bonding complex which broke up the weaker HR-HR hydrogen bonding interactions. The significant improvement of dispersability is depicted in Figure 8. After eight hours, pure nanoparticles [ZnO] were completely sedimentated in chloroform. The sedimentation of [ZnO-HR] was slightly reduced, while the final hybrids [ZnO-HR-CA] showed good stability in chloroform. As reference experiment to neglect any interactions of the cyanurate with [ZnO], the pure particles were added to a cyanurate solution, which had no impact on their stability.

**Figure 9:**
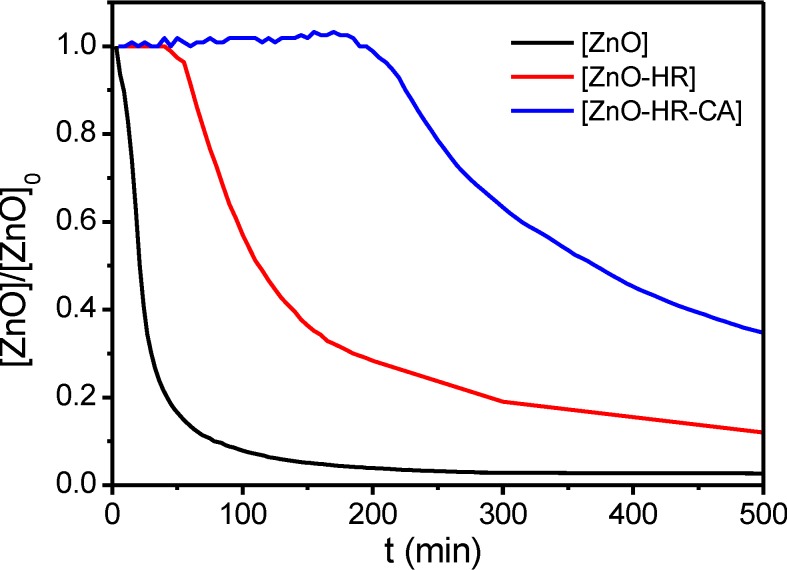
UV-Vis study of the sedimentation behaviour of [ZnO], [ZnO-HR] and [ZnO-HR-CA] in chloroform (plot of absorption maximum at λ = 360 nm *vs.* time (particle concentration: 0.01 wt %).

Time-dependent absorption measurements of the dispersions revealed the pronounced stability of the dispersion after noncovalent coating with the cyanurate (Figure 9). In the UV-Vis study, the absorption maximum of the ZnO nanorods at λ = 360 nm was plotted *vs.* time. A rapid decrease of absorption due to sedimentation was observed for [ZnO]. A slower decrease of [ZnO-HR] and the enhanced stability of [ZnO-HR-CA] confirmed the observations.

**Figure 10:**
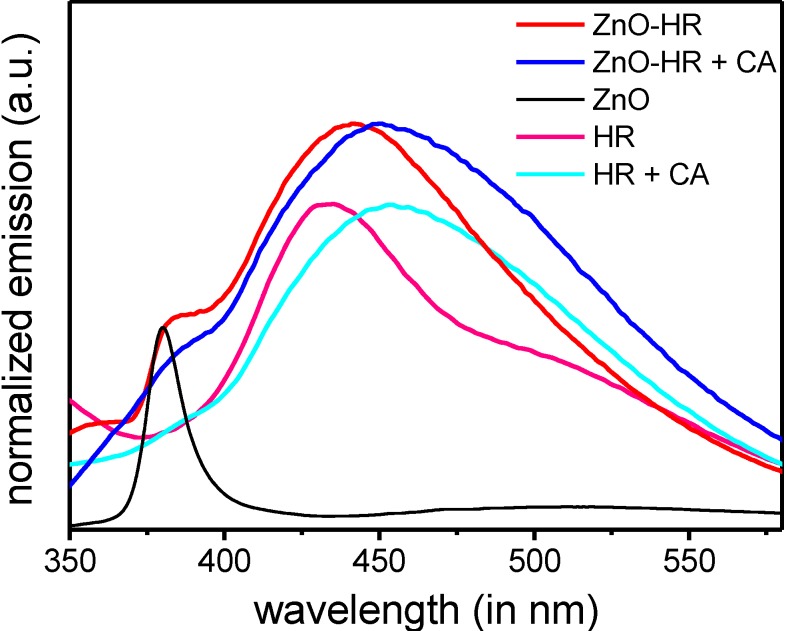
Emission spectra of Hamilton receptor **2** and [ZnO-HR], each before and after adding cyanurate **5** (exc. at λ = 300 nm).

IR spectra of functionalized nanorods [ZnO-HR] revealed similar absorption bands as the pure Hamilton receptor **4**. As characteristic band the N–H vibrations at *ṽ* = 3300 cm^−1^ are well pronounced. Upon addition of the cyanurate the hybrids [ZnO-HR-CA] are formed. As a result the N-H vibrations are broadened because of the formation of hydrogen bonds (see Supplementary Materials). Interactions of the Hamilton receptor with cyanurates were further monitored by fluorescence spectroscopy (Figure 10). Upon excitation of the Hamilton receptor **2** in chloroform at λ = 300 nm, a typical emission at λ = 437 nm was observed. This emission was shifted bathochromically by 17 nm after adding the cyanurate due to the formation of the hydrogen-bonding complex. Similarly, the coated nanoparticles [ZnO-HR] displayed an emission band at λ = 442 nm which was accounted to adsorbed Hamilton receptor **4**. After attachment of the second shell, the hybrid structures [ZnO-HR-CA] revealed a bathochromic shift, too. This shift to λ = 453 nm confirmed the formation of the supramolecular complex on the particle surface. A distinct maximum at λ = 377 nm within [ZnO] is originating from intrinsic formation and radiative recombination of excitons inside the ZnO bandstructure. This exciton band is slightly red shifted and superposed resuming a shoulder within the modified ZnO samples [ZnO-HR] and [ZnO-HR-CA]. The coupling experiments revealed that a successful noncovalent surface modification of the particles towards bilayer-coated hybrids [ZnO-HR-CA] was accomplished. The results outline the versatility of this construction principle of easily creating functional hybrid nanoparticles via noncovalent hydrogen bonding interactions, leaving the core architecture [ZnO-HR] unaffected.

## 3. Experimental Section

### 3.1. Materials and Methods

The ZnO nanorods [ZnO] were prepared according to procedures reported earlier [33]. In brief zinc oxide nanoparticles were prepared by precipitation from solutions of Zn(OAc)_2_ and KOH in methanol. To 125 mL of zinc acetate dihydrate solution, 65 mL KOH solution was dropped within 10 min at a refluxing temperature of 65 °C. Subsequent refluxing of the dispersions for 49 h led to the formation of ZnO nanorods. To remove remaining salt all the samples were washed three times with methanol. In a typical functionalization procedure 0.15 wt % solutions of ZnO nanorods in methanol (25 mL) were sonicated for two hours in different concentrations of catechol **4**. The functionalized particles [ZnO-HR] were centrifuged for isolation (3500 rpm; 15 min) and washed three times before analysis. The subsequent supramolecular coupling step was accomplished by addition of cyanuate **5** in a concentration of 1.0 mM to 0.15 wt % particle dispersions in chloroform. For analysis, the cyanurate-attached particles [ZnO-HR-CA] were centrifuged (3500 rpm; 15 min) and redispersed in chloroform.

The reagents and solvents were analytical grade reagents and used without further purification. The reactants 2,6-diaminopyridine, 3,3-dimethylbutyryl chloride, 5-nitroisophthalic acid, α,α-dichlorodiphenylmethane, protocatechuic acid ethyl ester, 8-bromooctanoic acid, cyanuric acid, zinc acetate dihydrate, potassium hydroxide, organic catalysts and solvents were purchased from Sigma Aldrich (St. Louis, MO, USA). Platinum(IV) oxide monohydrate for the hydrogenation of nitro-Hamilton receptor was obtained from Alfa-Aesar (Karlsruhe, Germany). Column chromatography was carried out on Silica gel 60 (particle size 0.04–0.063 mm; desactivated), purchased from Macherey-Nagel (Düren, Germany). NMR spectra were recorded with Bruker Avance 300 (300 MHz), Bruker Avance 400 (400 MHz), and Jeol EX 400 (400 MHz) spectrometers. FT-IR spectra were recorded on a Bruker Tensor 27 (ATR or ZnSe plate) spectrometer. A Varian Cary 5000 and Shimadzu UV-3102 PC were used for UV-Vis measurements. Fluorescence spectra were obtained from a Shimadzu RF-5301 PC. Mass spectrometry was carried out with a Shimadzu AXIMA Confidence (MALDI-TOF, matrices: 2,5-dihydroxybenzoic acid (dhb), *trans*-2-[3-(4-*tert*-butylphenyl)-2-methyl-2-propenylidene]malononitrile (dctb)). High resolution mass spectrometry (HRMS) was performed on a Bruker microTOF II focus and a Bruker maXis 4G. For determination of the zeta potentials, a Zetasizer Nano series ZEN3600 (Malvern Instruments, Herrenberg, Germany) was used. A 633 nm laser was taken to detect particle size distribution. TGA and TGA-MS experiments were carried out on a TGA/SDTA 851e (Mettler Toledo, Columbus, OH, USA), a TG-209 F1 Libra (Netzsch, Selb, Germany), and a Skimmer STA 409 CD (Netzsch) with nitrogen and oxygen as protective and carrier gas. The centrifuge was a Heraeus Multifuge X1R (Thermo scientific, Waltham, MA, USA).

### 3.2. Synthesis

3,4-Diphenylmethylenedioxyprotoctechuic acid (**1**) was synthesized starting with Ethyl 3,4-dihydroxybenzoate in two steps following a literature procedure [37]. The amine-Hamilton receptor (**2**) and its precursors were synthesized according to Dirksen *et al.* [16] The compounds (**3**) and (**4**) were prepared as following:

*N*^1^,*N*^3^‑*bis*(6-(3,3-dimethylbutanamido)pyridine-2-yl)-5-(2,2-diphenylbenzo[d][1,3]di-oxole-5-carboxamido)isophthalamide (**3**): Under dry conditions 3,4-diphenylmethyleneoxyprotocatechuic acid (**1**) (130 mg; 0.42 mmol) was dissolved in 20 mL dry DMF and cooled to 0 °C. Subsequently, 1-hydroxybenzotriazole (110 mg; 0.83 mmol) was added and the solution was stirred for 15 min at 0 °C. Then 1-ethyl-3-(3-dimethylaminopropyl)carbodiimide (160 mg; 0.83 mmol) was added and the suspension was stirred for another 45 min at 0 °C. Amine-Hamilton receptor (**2**) (700 mg; 1.25 mmol) was added and the solution was stirred for 20 h at room temperature. The raw product was purified by column chromatography (SiO_2_, DCM/EtOAc 2:1). After evaporation of the solvent, compound **3** was obtained as a white solid (296 mg; 0.34 mmol; 82%). UV-Vis (MeOH): λ = 301 nm; IR (ATR): *ṽ*_max_ = 3055, 2951, 2862, 1666, 1622, 1585, 1492, 1443, 1288, 1246, 1015, 798, 752, 698 cm^−1^; ^1^H-NMR (CDCl_3_, 400 MHz, rt): δ [ppm] = 8.97 (br, 3H, NH), 8.45 (br, 2H, cat-CH), 8.24 (br, 2H, NH), 7.96 (s, 1H, cat-CH), 7.87 (d, ^3^*J* = 8.06 Hz, 2H, py-CH), 7.77 (d, ^3^*J* = 7.94 Hz, 2H, py-CH), 7.60 (m, 2H, cat-CH), 7.53 (m, 6H, Ph-CH & py-CH), 7.35 (m, 6H, Ph-CH), 6.89 (d, ^3^*J* = 8.42 Hz, 1H, cat-CH), 2.42 (s, 4H, CH_2_), 1.11 (s, 18H, CH_3_); ^13^C-NMR (CDCl_3_, 100.5 MHz, RT): δ [ppm] = 171.76 (1C, C=O), 166.34 (1C, C=O), 163.64 (1C, C=O), 151.01 (1C, ^q^C), 150.18 (1C, ^q^C), 148.78 (1C, ^q^C), 147.98 (1C, ^q^C), 140.46 (1C, ^q^C), 139.41 (1C, Ph-CH), 135.06 (1C, ^q^C), 129.47 (1C, Ph-CH), 128.39 (1C, Ph-CH), 127.66 (1C, ^q^C), 126.20 (1C, Ph-CH), 122.68 (1C, Ph-CH), 121.80 (1C, Ph-CH), 118.49 (1C, Ph-CH), 115.73 (1C, ^q^COO), 110.70 (1C py-CH), 109.27 (1C, py-CH), 108.50 (1C, Ph-CH), 108.01 (1C, Ph-CH), 50.93 (2C, ^q^C(CH_3_)_3_), 31.49 (2C, CH_2_), 29.84 (6C, CH_3_). MS (MALDI-TOF, dhb): *m*/*z* = 882 [M + Na]^+^; HRMS (ESI-TOF, acetonitrile/methanol) found: *m*/*z* = 882.3573; calcd: 882.3585 [M + Na]^+^.

5-(3,4-Dihydroxybenzamido)-*N*^1^,*N*^3^-*bis*(6-(3,3-dimethylbutanamido)pyridin-2-yl)isophthalamide (**4**): *N*^1^,*N*^3^-*bis*(6-(3,3-dimethylbutanamido)pyridin-2-yl)-5-(2,2-diphenylbenzo[d][1,3]dioxole-5-carboxamido)isophthalamide (**3**) (200 mg; 0.23 mmol) was dissolved in 15 mL trifluoroacetic acid and the solution was stirred for 24 h at room temperature. The TFA was removed *in vacuo*. Recrystallization from pentane gave pure product as white solid (159 mg; 0.23 mmol; 99%). UV-Vis (MeOH): λ = 228, 302 nm; IR (ATR): *ṽ*_max_ = 3280, 3081, 2958, 2874, 1670, 1649, 1621, 1585, 1555, 1491, 1441, 1293, 1187, 1118, 1040, 1018, 795, 752, 696 cm^−1^; ^1^H-NMR (THF-*d*_8_, 400 MHz, RT): δ [ppm] = 9.67 (br, 1H, NH), 9.49 (br, 2H, NH), 9.27 (br, 2H, NH), 8.64 (br, 2H, Ph-CH), 8.18 (s, 1H, Ph-CH), 8.00 (m, 4H, py-CH), 7.72 (t, ^3^*J* = 8.06 Hz, 2H, py-CH), 7.49 (s, 1H, cat-CH), 7.43 (d, ^3^*J* = 8.30 Hz, cat-CH) 6.81 (d, ^3^*J* = 8.30 Hz, 1H, cat-CH), 5.99 (br, 2H, OH), 2.28 (s, 4H, CH_2_), 1.09 (s, 18H, CH_3_); ^13^C-NMR (THF-*d*_8_, 100.5 MHz, RT): δ [ppm] = 170.95 (2C, C=O), 166.42 (1C, C=O), 165.34 (2C, C=O), 150.16 (2C, ^q^C), 146.16 (2C, ^q^C), 141.68 (1C, COH), 136.44 (2C, py-CH), 132.83 (1C, COH), 130.51 (1C, ^q^C), 128.91 (2C, ^q^C), 127.07 (1C, ^q^C), 122.54 (2C, Ph-CH), 121.70 (1C, Ph-CH), 120.41 (1C, cat-CH), 115.82 (1C, cat-CH), 115.32 (1C, cat-CH), 50.68 (2C, ^q^C(CH_3_)_3_), 31.64 (2C, CH_2_), 29.99 (6C, CH_3_); MS (MALDI-TOF, dhb): *m*/*z* = 696 [M]^+^; HRMS (ESI-TOF, acetonitrile/methanol) found: *m*/*z* = 718.2953; calcd: 718.2959 [M + Na]^+^.

The synthesis of Ethyl 8-bromooctanoate and Ethyl 8-(2,4,6-trioxo-1,3,5-triazinan-1-yl)octanoate (**5**) was carried out following a similar procedure recently described by us [30]. The synthetic procedure and characterization is depicted in ESI.

## 4. Conclusions

In conclusion, we described a versatile concept for the construction of nano hybrid architectures through noncovalent grafting of cyanurates to Hamilton receptor modified nanoparticle surfaces. The approach combines unique characteristics of stable covalent surface attachment with advantages of noncovalent supramolecular chemistry. A tailor-designed Hamilton receptor **4** covalently equipped with a catechol anchor group was synthesized and grafted to freshly prepared ZnO nanorods [ZnO]. A detailed functionalization study towards monolayer grafted particles [ZnO-HR] revealed a maximum monolayer grafting density of 1.1 molecules per nm^2^. These building blocks were accessible for noncovalent attachment of cyanurates **5** to yield the hybrid architectures [ZnO-HR-CA], which displayed enhanced dispersability in apolar solvents. The results outline the versatility of this unique construction principle and open up an exciting option for the formation of well-oriented organic-inorganic hybrid architectures for applications in nano-electronics and biotechnology. Further extension and elaboration of such assembly protocols including variation of the inorganic cores (e.g., TiO_2_, SiO_2_, Au) and cyanurate-functionalities (e.g., chromophores; semiconductors) are currently underway in our laboratory.

## Supplementary Materials

Electronic Supplementary Information (ESI) available: details of synthetic procedures, additional characterization and grafting density calculations. Supplementary materials can be found at http://www.mdpi.com/1422-0067/16/04/8186/s1.

## References

[1] Whitesides G.M., Grzybowski B. (2002). Self-Assembly at all scales. Science.

[2] Ikkala O., ten Brinke G. (2002). Functional materials based on self-assembly of polymeric supramolecules. Science.

[3] Lehn J.-M. (2002). Toward self-organization and complex matter. Science.

[4] Pachón L.D., Rothenberg G. (2008). Transition-metal nanoparticles: Synthesis, stability and the leaching issue. Appl. Organomet. Chem..

[5] Garnweitner G., Hashim A.A. (2012). *In-situ vs.* post-synthetic stabilization of metal oxide nanoparticles. The Delivery of Nanoparticles.

[6] Segets D., Marczak R., Schäfer S., Paula C., Gnichwitz J.-F., Hirsch A., Peukert W. (2011). Experimental and theoretical studies of the colloidal stability of nanoparticles—A general interpretation based on stability maps. ACS Nano.

[7] Shenhar R., Rotello V. (2003). Nanoparticles: Scaffolds and building blocks. Acc. Chem. Res..

[8] Gnichwitz J.-F., Marczak R., Werner F., Lang N., Jux N., Guldi D.M., Peukert W., Hirsch A. (2010). Efficient synthetic access to cationic dendrons and their application for ZnO nanoparticles surface functionalization: New building blocks for dye-sensitized solar cells. J. Am. Chem. Soc..

[9] Mann S., Shenton W., Li M., Connolly S., Fitzmaurice D. (2000). Biologically programmed nanoparticle assembly. Adv. Mater..

[10] Boal A.K., Ilhan F., DeRouchey J.E., Thurn-Albrecht T., Russell T.P., Rotello V.M. (2000). Self-assembly of nanoparticles into structured spherical and network aggregates. Nature.

[11] Tan L.H., Xing H., Lu Y. (2014). DNA as a powerful tool for morphology control, spatial positioning and dynamic assembly of nanoparticles. Acc. Chem. Res..

[12] Niemeyer C.M. (2001). Nanoparticles, proteins, and nucleic acids: Biotechnology meets materials science. Angew. Chem. Int. Ed. Engl..

[13] Chang S.-K., Hamilton A.D. (1988). Molecular recognition of biologically interesting substrates: Synthesis of an artificial receptor for barbiturates employing six hydrogen bonds. J. Am. Chem. Soc..

[14] Chang S.-K., Engen D.V., Fan E., Hamilton A.D. (1991). Hydrogen bonding and molecular recognition: Synthetic, complexation, and structural studies on barbiturate binding to an artificial receptor. J. Am. Chem. Soc..

[15] Dethlefs C., Eckelmann J., Kobarg H., Weyrich T., Brammer S., Näther C., Lüning U. (2011). Determination of binding Constants of hydrogen-bonded complexes by ITC, NMR CIS, and NMR diffusion experiments. Eur. J. Org. Chem..

[16] Dirksen A., Hahn U., Schwanke F., Nieger M., Reek J.N.H., Vögtle F., de Cola L. (2004). Multiple Recognition of barbiturate guests by hamilton-receptor-functionalized dendrimers. Chem. Eur. J..

[17] Franz A., Bauer W., Hirsch A. (2005). Complete self-assembly of discrete supramolecular dendrimers. Angew. Chem. Int. Ed. Engl..

[18] Hager K., Hartnagel U., Hirsch A. (2007). Supramolecular dendrimers self-assembled from dendritic fullerene ligands and a homotritopic hamilton receptor. Eur. J. Org. Chem..

[19] Binder W.H., Kluger C., Straif C.J., Friedbacher G. (2005). Directed nanoparticle binding onto microphase-separated block copolymer thin films. Macromolecules.

[20] Grimm F., Ulm N., Gröhn F., Düring J., Hirsch A. (2011). Self-assembly of supramolecular architectures and polymers by orthogonal metal complexation and hydrogen-bonding motifs. Chem. Eur. J..

[21] Zirbs R., Kienberger F., Hinterdorfer P., Binder W.H. (2005). Directed assembly of Au nanoparticles onto planar surfaces via multiple hydrogen bonds. Langmuir.

[22] Motesharei K., Myles D.C. (1998). Molecular recognition on functionalized self-assembled monolayers of alkanethiols on gold. J. Am. Chem. Soc..

[23] Ema T., Okuda K., Watanabe S., Yamasaki T., Minami T., Esipenko N.A., Anzenbacher P. (2014). Selective anion sensing by chiral macrocyclic receptors with multiple hydrogen-bonding sites. Org. Lett..

[24] Tron A., Thornton P.J., Rocher M., Jacquot de Rouville H.-P., Desvergne J.-P., Kauffmann B., Buffeteau T., Cavagnat D., Tucker J.H., McClenaghan N.D. (2014). Formation of a hydrogen-bonded barbiturate [2]-rotaxane. Org. Lett..

[25] Tecilla P., Dixon R.P., Slobodkin G., Alavi D.S., Waldeck D.H., Hamilton A.D. (1990). Hydrogen-bonding self-assembly of multichromophore structures. J. Am. Chem. Soc..

[26] Dirksen A., Kleverlaan C.J., Reek J.N.H., de Cola L. (2005). Ultrafast photoinduced electon transfer within a self-assembled donor-acceptor system. J. Phys. Chem. A.

[27] Würthner F., Schmidt J., Stolte M., Wortmann R. (2006). Hydrogen-bond-directed head-to-tail orientation of dipolar merocyanine dyes: A strategy for the design of electrooptical materials. Angew. Chem. Int. Ed. Engl..

[28] Wessendorf F., Gnichwitz J.-F., Sarova G.H., Hager K., Hartnagel U., Guldi D.M., Hirsch A. (2007). Implementation of a hamilton-receptor-based hydrogen-bonding motif toward a new electron donor-acceptor prototype: Electron *vs.* energy transfer. J. Am. Chem. Soc..

[29] Gnichwitz J.-F., Wielopolski M., Hartnagel K., Hartnagel U., Guldi D.M., Hirsch A. (2008). Cooperativity and tunable excited state deactivation: Modular self-assembly of depsipeptide dendrons on a hamilton receptor modified porphyrin platform. J. Am. Chem. Soc..

[30] Bosch S., Zeininger L., Hauke F., Hirsch A. (2014). A supramolecular approach for the facile solubilization and separation of covalently functionalized single-walled carbon nanotubes. Chem. Eur. J..

[31] Pujari S.P., Scheres L., Marcelis A.T. M., Zuilhof H. (2014). Covalent surface modification of oxide surfaces. Angew. Chem. Int. Ed. Engl..

[32] Schönamsgruber J., Zeininger L., Hirsch A. (2014). Grafting perylenes to ZnO nanoparticles. Chem. Eur. J..

[33] Voigt M., Klaumünzer M., Thiem H., Peukert W. (2010). Detailed analysis of the growth kinetics of ZnO nanorods in methanol. J. Phys. Chem. C.

[34] Sun Y., Rogers J.A. (2007). Inorganic semiconductors for flexible electronics. Adv. Mater..

[35] Schaeffler G., Fuhr O., Fenske D., Lehn J.-M. (2014). Self-assembly of a highly organized, hexameric supramolecular architecture: Formation, structure and properties. Chem. Eur. J..

[36] Portilla L., Halik M. (2014). Smoothly tunable surface properties of aluminum oxide core-shell nanoparticles by a mixed-ligand approach. ACS Appl. Mater. Interfaces.

[37] Iacazio G., Périssol C., Faure B. (2000). A new tannase substrate for spectrophotometric assay. J. Microbiol. Methods.

