# Supplementary material for: Surface Modification of ZnO Nanorods with Hamilton Receptors
Source: Int J Mol Sci. 2015 Apr 13;16(4):8186–200. doi: 10.3390/ijms16048186 (PMC4425075; doi:10.3390/ijms16048186)
Supplement: Supplementary file 1 [file ijms-16-08186-s001.pdf]

Supplementary Information

(1) Synthesis and Characterization

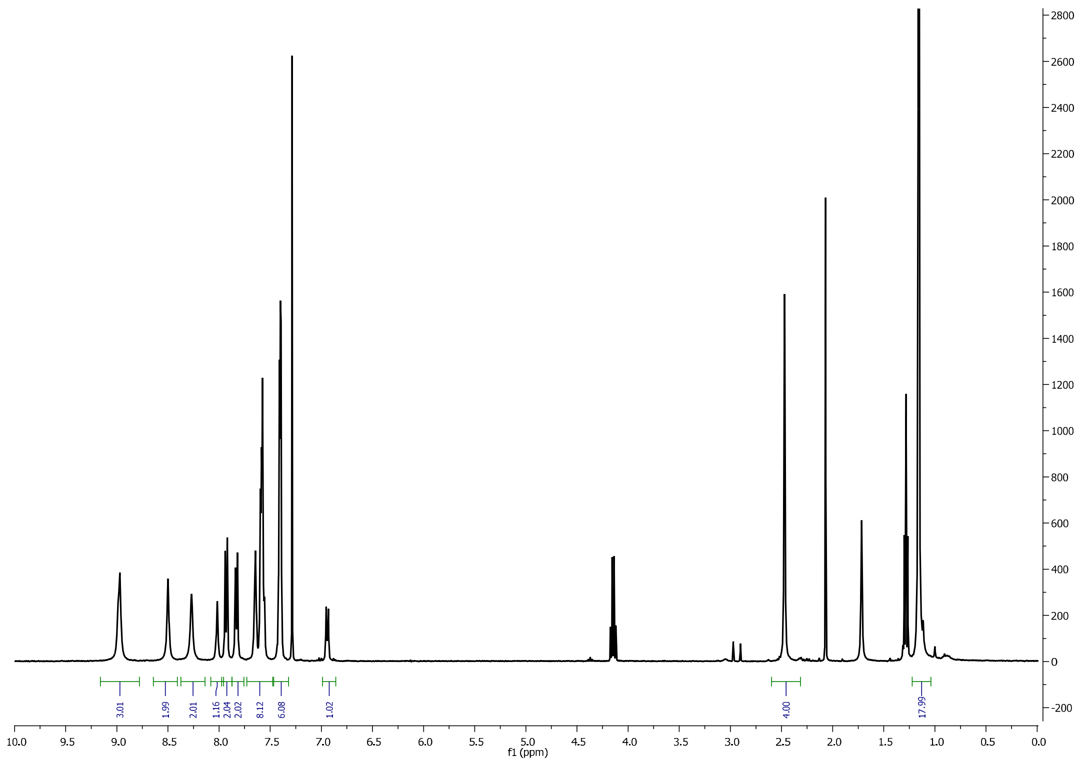

Figure S1. ^{1}H-NMR of **3** (400 MHz; $CDCl_3$; rt).

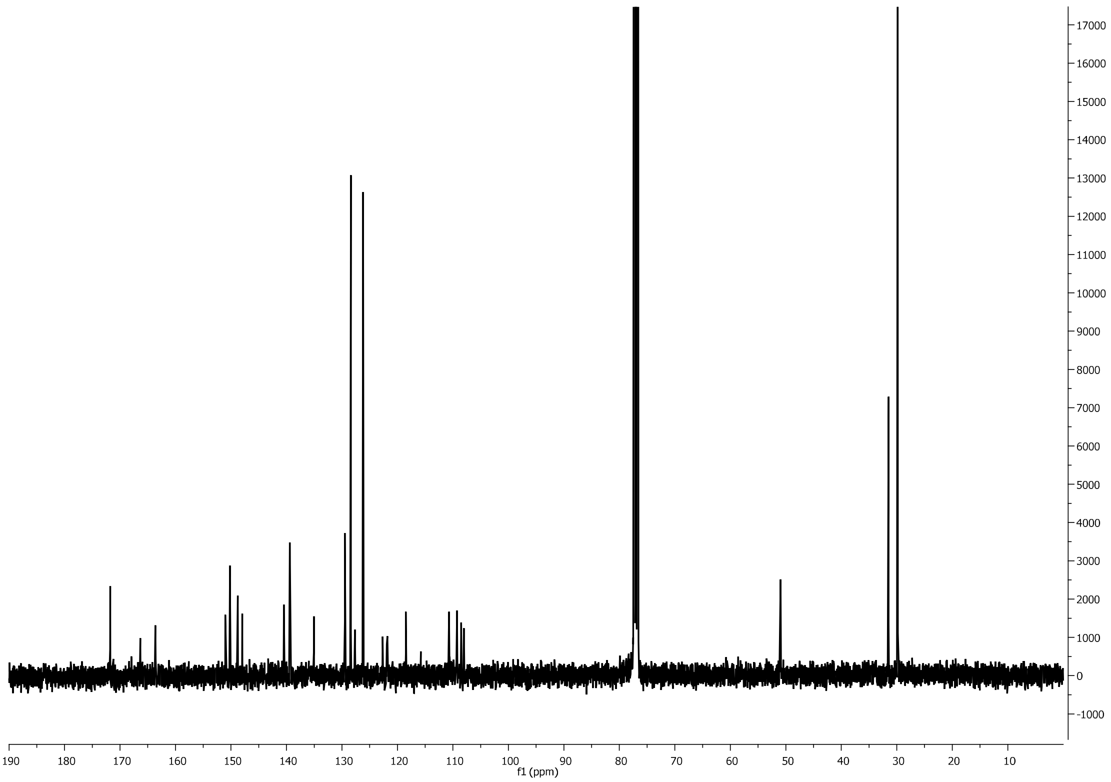

Figure S2. ^{13}C-NMR of **3** (100 MHz; $CDCl_3$; rt).

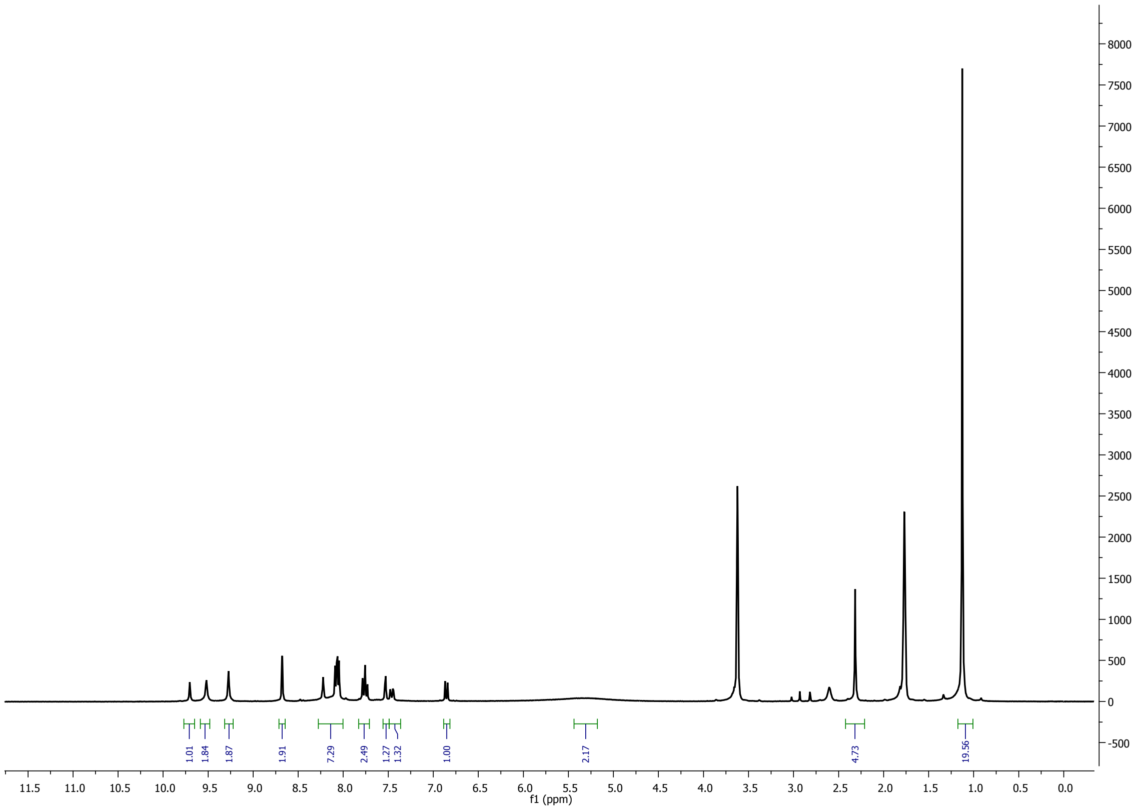

Figure S3. ^{1}H-NMR of **4** (400 MHz; THF-*d*8; rt).

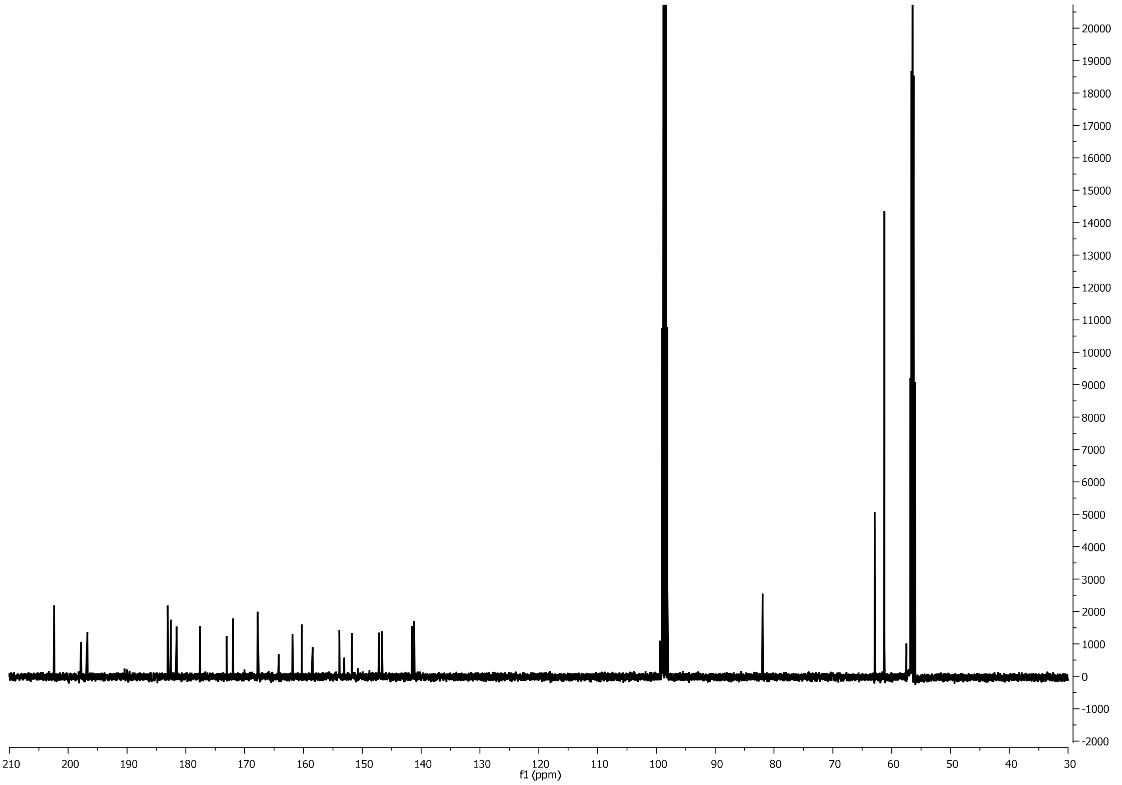

Figure S4. ^{13}C-NMR of **4** (100 MHz; THF-*d*8; rt).

Synthesis and Characterization of **5**

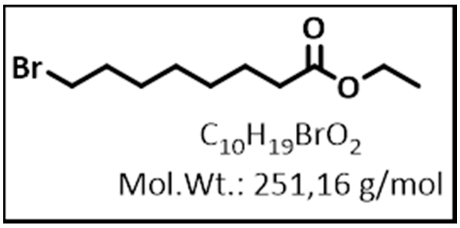

0.90 g (4.00 mmol) 8-Bromooctanoic acid was dissolved in 30 mL ethanol. Then 3 mL sulfuric acid were added. The mixture was heated at reflux temperature for 12 h and allowed to cool down to rt. 50 mL Water was added and the product extracted with diethyl ether. The organic phase was washed with brine and dried with $MgSO_4$. Evaporation of the solvent gave Ethyl 8-bromooctanoate. Yield: 0.96 g (3.80 mmol; 95%). ^{1}H-NMR ($CDCl_3$, 400 MHz, rt): δ [ppm] = 1.23 (t, ^3J = 7.1 Hz, 3H, CH_3), 1.30 (m, 6H, CH_2), 1.60 (m, 2H, CH_2), 1.82 (m, 2H, CH_2), 2.26 (t, ^3J = 7.4 Hz, 2H, C=OCH_2), 3.37 (t, ^3J = 6.8 Hz, 2H, BrCH_2), 4.09 (q, ^3J = 7.1 Hz, 2H, CH_3-CH_2); MS (MALDI; dhb): *m*/*z* = 251 $[M]^+$.

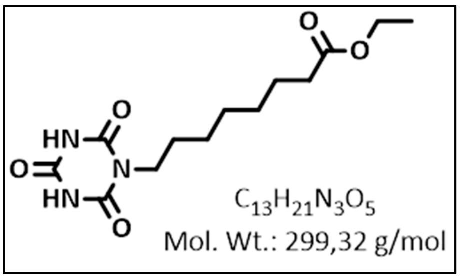

A suspension of 1.00 g (3.98 mmol) Ethyl 8-bromooctanoate, 1.03 g (7.96 mmol) cyanuric acid and 0.59 mL (3.98 mmol) DBU in 30 mL dry DMF was heated at 70 °C under inert gas atmosphere for 20 h. After cooling, the yellow solution was poured into 100 mL cold water. The resulting white precipitate was extracted with 3 × 100 mL EtOAc. The organic phase was dried over $MgSO_4$. Then, the raw product was adsorbed on silica and purified by flash column chromatography (SiO_2, cyclohexane/ethyl acetate 1:1). The desired compound was obtained as white solid. Yield: 0.94 g (2.79 mmol; 79%); ^{1}H-NMR ($CDCl_3$, 400 MHz, rt): δ [ppm] = 1.23 (t, ^3J = 7.1 Hz, 3H, CH_3), 1.30 (m, 6H, CH_2), 1.59 (m, 4H, CH_2), 2.26 (t, ^3J = 7.4 Hz, 2H, C=OCH_2), 3.81 (t, ^3J = 6.8 Hz, 2H, CyAc-CH_2), 4.08 (q, ^3J = 7.1 Hz, 2H, CH_3–CH_2), 9.14 (br, 2H, N-H). ^{13}C-NMR ($CDCl_3$, 100 MHz, rt): δ [ppm] = 173.94 (1C, O*C*=O), 149.06 (1C,NH*C*=ONH), 147.97 (1C, N*C*=O), 60.26 (1C, *C*H_2CH_3), 41.97 (1C, *C*H_2), 34.27 (1C, *C*H_2), 28.90 (1C, *C*H_2), 28.76 (1C, *C*H_2), 27.64 (1C, *C*H_2), 26.34 (1C, *C*H_2), 24.79 (1C, *C*H_2), 14.22 (1C, *C*H_3); MS (MALDI, om): *m*/*z* = 299 $[M]^+$; IR (ATR): $\tilde{\nu}_{max}$ [cm^{-1}] = 3204, 3083, 2931, 2861, 1764, 1729, 1681, 1465, 1415, 1373, 1176, 792, 761, 547.

(2) Supramolecular Coupling

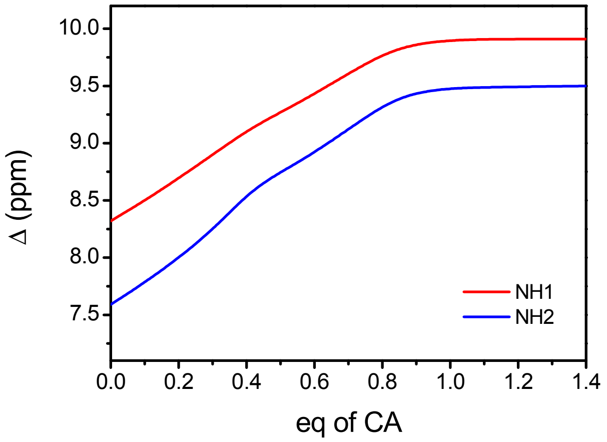

Figure S5. Hyp-NMR fitted binding isotherm of 2 and 5, derived from 1H-NMR-titration data.

(3) Surface Functionalization

BET analysis ZnO nanorods
Quantachrome NovaWin—Data Acquisition and Reduction for NOVA instruments
©1994–2007, Quantachrome Instruments—version 10.0

**Analysis**		**Report**			
Sample Desc	1	Comment	120 °C, 2 h		
Sample weight	0.185 g	Sample Volume	0 cc		
Outgas Time	2.0 h	Outgas Temp.	120.0 C		
Analysis gas	Nitrogen	Bath Temp.	77.3 K		
Press. Tolerance	0.100/0.100 (ads/des)	Equil time	60/60 s (ads/des)	Equil timeout	240/240 s (ads/des)
Analysis Time	92.2 mi	Cell ID	0		
Adsorbate	Nitrogen	Temperature	77.350 K		
Molec. Wt.	28.013 g	Cross Section	16.200 Å^2	Liquid Density	0.808 g/cc

**Relative/Pressure (P/Po)**	**Volume @ STP (cc/g)**	**1/[W((Po/P) − 1)]**
4.97760×10^{-2}	3.1309	13.387
1.17470×10^{-1}	4.2780	24.895
1.76212×10^{-1}	4.9801	34.366
2.38835×10^{-1}	5.5625	45.133
2.99066×10^{-1}	6.0434	56.488

**BET Summary**
Slope = 171.668
Intercept = 4.594
Correlation coefficient, r = 0.999635
C constant = 38.370
Surface Area = 19.758 m^2/g

TGA measurements of functionalized ZnO nanorods

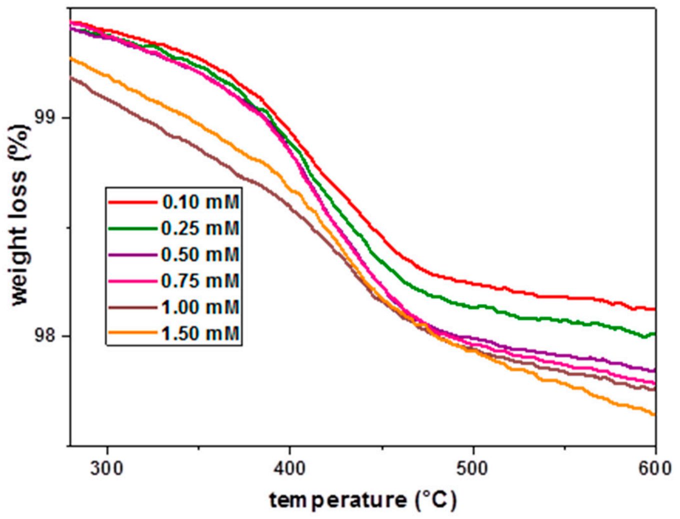

Figure S6. TGA of [ZnO-HR], functionalized with different concentrations of HR **4**. (TGA performed under N_2 and O_2 gas flow; heat rate 10 K/min).

Table S1. Calculation of grafting densities of [ZnO-HR] from TGA data: grafting density $= \left(\frac{\text{wt}}{100-\text{wt}}\right)\left(\frac{6.022\times10^{23}}{Mw \times \text{SSA}}\right)$. wt: weight loss from TGA (%); *M*w of **4**; SSA: 19.76 m^2/g.

Concentration (mM)	Organic Mass Loss (TGA) (%)	Grafting Density (molecules/nm^2)
0.10	1.37	0.608
0.25	1.91	0.839
0.50	2.21	0.967
0.75	2.47	1.082
1.00	2.55	1.118
1.50	2.62	1.151

Table S2. Theoretical grafting density of adsorbates on ZnO nanorods (calculated for wet-chemical functionalization in MeOH-density = 0.8 g/mL and with SSA = 19.76 m^2/g).

Grafting Density (molecules/nm^2)	1	2	3	4	5	6	7
molecules needed for full coverage (E+18) (per 25 mL of 0.15 wt% − solution)	0.5928	1.1856	1.7784	2.3712	2.9640	3.5568	4.1496

Table S3. Calculation of amount of employed molecules for wet-chemical surface functionalization.

Concentration (mM)	Molecules Employed for Functionalization (E+18)	Concentration (mM)	Molecules Employed for Functionalization (E+18)
0.025	0.3764	1.00	15.055
0.05	0.7528	1.50	22.583
0.10	1.5055	2.00	30.111
0.20	3.0111	3.00	45.166
0.25	3.7638	6.00	90.332
0.30	4.5166	12.00	180.66
0.60	9.0332	25.00	376.38

Interpretation of UV-Vis data:

The grafting density was calculated from UV-vis by employing the following formula:

$$\theta = \left(\frac{(\mathrm{Abs}_{302\mathrm{nm}} - \mathrm{Abs}_{302\mathrm{nm}}(\mathrm{ZnO})) \times V}{\varepsilon \times d}\right) \times \left(\frac{6.022 \times 10^{23}}{SA}\right)$$

$\mathrm{Abs}_{302\mathrm{nm}}$: measured absorption; $\mathrm{Abs}_{302\mathrm{nm}}(\mathrm{ZnO})$: bandgap absorption of ZnO; E: extinction coefficient of Hamilton receptor 4 at 302 nm: 54,861 $\mathrm{L \cdot mol^{-1} \cdot cm^{-1}}$; D: diameter UVvis cuvette: 1 cm; V: volume in UVvis cuvette: 2 mL; SA: surface area of ZnO nanorods in 2 mL UV-solution (~2.4 µg·rods): $47.42 \times 10^{12}\ \mathrm{nm}^2$.

Calculation of Langmuir Adsorption isotherm

Formula Langmuir isotherm:

$$\theta = \frac{c \times \theta\ \mathrm{max}}{K(\mathrm{des}) + c}$$

Linear form Langmuir isotherm:

$$\frac{1}{\theta} = \frac{K(\mathrm{des})}{(\theta\ \mathrm{max})} + \frac{1}{\theta\ \mathrm{max}}$$

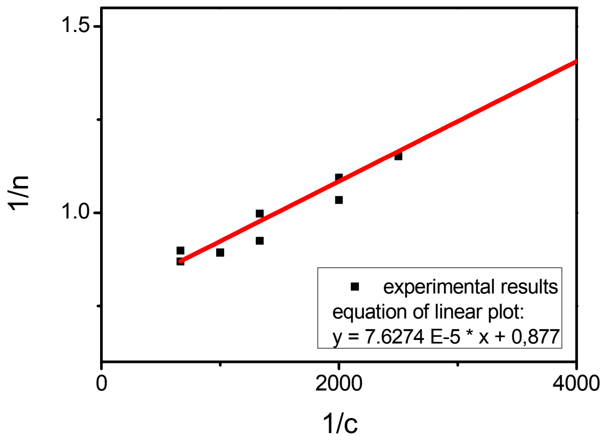

Figure S6. Linear plot of experimental results with corresponding equation.

Calculation of maximum monolayer grafting density:

$$\theta\ (\max) = \frac{1}{0.877} = 1.14025$$

Calculation of the adsorption constant for the grafting of ZnO nanorods with catechol 4 in methanol:

$$K(ads) = 1/(7.6274 \times 10^{-5} \times \theta max) = 11{,}498$$

Insertion of K(ads) and n(max) leads to calculated Langmuir isotherm:

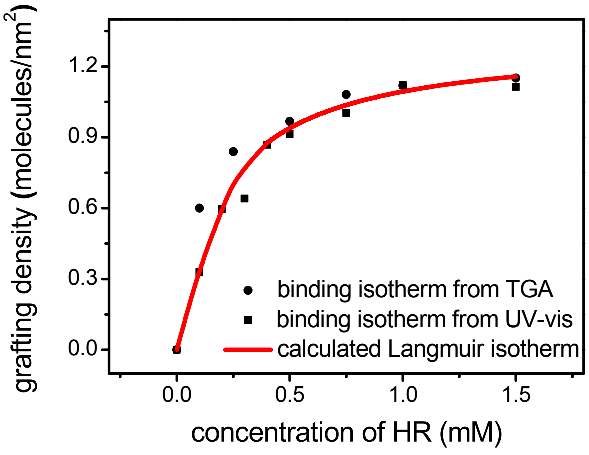

Figure S7. Calculated Langmuir isotherm.

(4) Hybrid formation

Table S4. DLS-measurements of ZnO, ZnO-HR and ZnO-HR-CA.

Sample (Measured in Chloroform)	Hydrodynamic Diameter (from DLS) (in nm)	Standard Deviation (in nm)
[ZnO]	141.9	4.9
[ZnO-HR]	158.5	5.0
[ZnO-HR-CA]	167.0	3.8

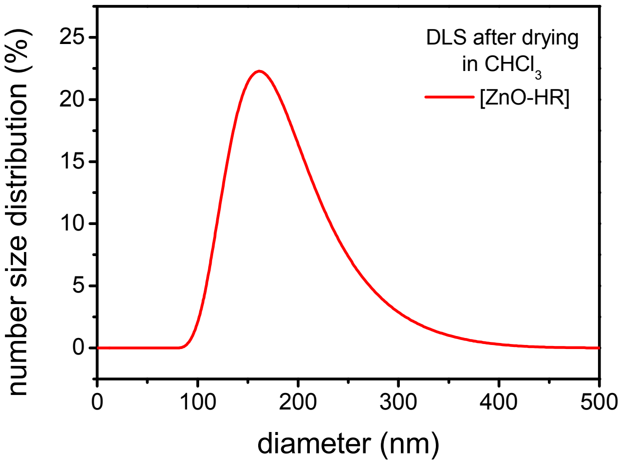

Figure S8. DLS-measurement of [ZnO-HR] after centrifugation and drying (80 °C) for 24 h.

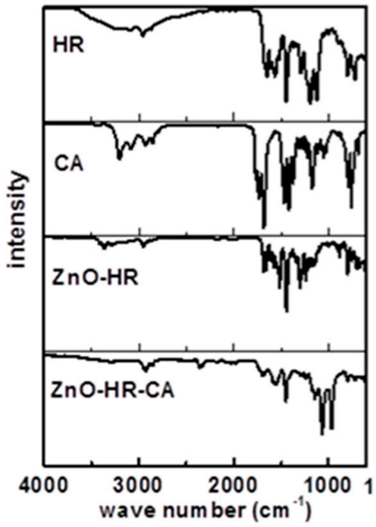

Figure S9. IR spectra of ZnO, ZnO-HR and ZnO-HR-CA.

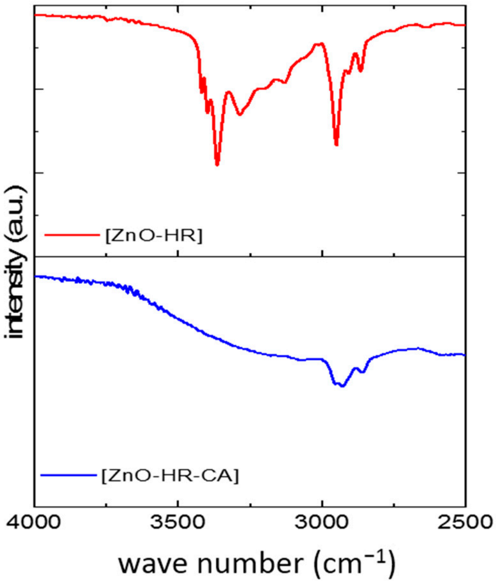

Figure S10. IR spectra ZnO-HR and ZnO-HR-CA; magnified region displays broadening of the NH-IR-vibrations upon H-bond formation.
